# Thermomechanical and microhardness data of melamine-formaldehyde-based self-healing resin film able to undergo reversible crosslinking via Diels-Alder reaction

**DOI:** 10.1016/j.dib.2020.106559

**Published:** 2020-11-21

**Authors:** Katharina Urdl, Petra Christöfl, Stephanie Weiss, Andreas Kandelbauer, Uwe Müller, Wolfgang Kern

**Affiliations:** a Kompetenzzentrum Holz (Wood K Plus), Altenberger Straße 69, A-4040 Linz; c/o: Kompetenzzentrum Holz (Wood K Plus), Wood Carinthian Competence Center (W3C), Klagenfurterstraße 87-89, 9300 Sankt Veit an der Glan, Austria; b Polymer Competence Center Leoben GmbH, A-8700 Leoben, Austria; c Chair in Chemistry of Polymeric Materials, Montanuniversitaet Leoben, Otto Glöckel-Straße 2/IV, A-8700 Leoben, Austria; d Reutlingen University, Lehr- und Forschungszentrum Process Analysis & Technology (PA&T), School of Applied Chemistry, Alteburgstraße 150, D-72762 Reutlingen, Germany

**Keywords:** Self-healing, Melamine resin film, Decorative laminates, Diels-Alder, nanoindentation, Dynamic-load thermomechanical analysis

## Abstract

The data presented in this article characterize the thermomechanical and microhardness properties of a novel melamine-formaldehyde resin (MF) intended for the use as a self-healing surface coating. The investigated MF resin is able to undergo reversible crosslinking via Diels Alder reactive groups. The microhardness data were obtained from nanoindentation measurements performed on solid resin film samples at different stages of the self-healing cycle. Thermomechanical analysis was performed under dynamic load conditions. The data provide supplemental material to the manuscript published by Urdl et al. 2020 (http://doi.org/10.1016/j.eurpolymj.2020.109601, [1]) on the self-healing performance of this resin, where a more thorough discussion on the preparation, the properties of this coating material and its application in impregnated paper-based decorative laminates can be found [1].

## Specifications Table

**Table:** 

Subject	Material Science, Polymer Applied Science
Specific subject area	Self-Healing Melamine formaldehyde resin synthesis and properties
Type of data	Table Graph
How data were acquired	Dynamic Load Thermomechanical Analysis (DL-TMA) Nanoindentation measurement
Data format	Raw
Parameters for data collection	A novel melamine-formaldehyde based resin surface film containing Diels Alder reactive functional groups was prepared and analyzed for its self-healing properties after damaging the film by scratching. The resin film was investigated as a cured solid resin film. Various heating cycles were covered by the applied temperature program.
Description of data collection	The cured MF-resin able to undergo self-healing due to Diels Alder functional groups was prepared as described in Urdl et al. 2020 [1]. The cured resin films were analyzed with DL-TMA and nanoindentation in order to characterize the mechanical performance of the MF resin when subjected to several self-healing cycles.
Data source location	Kompetenzzentrum Holz (Wood K plus), Sankt Veit an der Glan, Austria
Data accessibility	All raw data are available in this article.
Related research article	These data are supplementary to the article: K. Urdl, S. Weiss, P. Christöfl, A. Kandelbauer, U. Müller, W. Kern, Diels-Alder modified self-healing melamine resin, Eur. Polym. J. 127 (2020), 109601. https://doi.org/10.1016/j.eurpolymj.2020.109601

## Value of the Data

• The data allow a better understanding of the relationships described in the main paper and allow discussion of the properties of all investigated samples. They provide insight into the thermal-mechanical properties as well as the microhardness profiles of the produced melamine formaldehyde resin. It is important to understand the regeneration of its mechanical performance after undergoing several self-healing cycles.
• Engineers and scientists who are working in the field of self-healing phenomena will be interested in these data, especially if they focus on the self-amending properties of thermosetting materials. The data are especially relevant to surface scientists, engineers who work in the field of coatings, laminates and engineered wood surface finishes.
• The data can be compared to those of other resin films with self-healing properties and can be a reference for the development of resins formulations to be applied for laminate surfaces.
• From the DL TMA measurements the shrinkage and expansion behavior of the self-healable MF resin is deduced. This indicates action of reversible or irreversible chemical reactions. Shrinkage indicates irreversible chemical reaction. A detailed discussion of the thermoanalytical data is found in [1]
• From the nanoindenter profiles it is seen that the samples become increasingly stiffer as the number of self-healing cycles increases (i.e. the E modulus increases). A detailed discussion of the nanoindenter data is found in [1]

## 1 Data Description

**Fig. 1:**
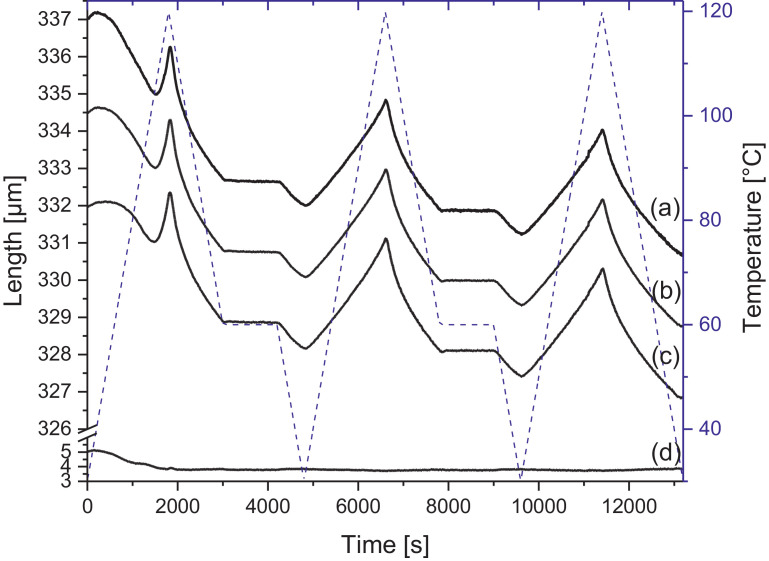
DL-TMA traces; (a) upper envelope; (b) average change in elongation; (c) lower envelope; (d) amplitude level; temperature programme is plotted as blue dashed line.

**Fig. 2:**
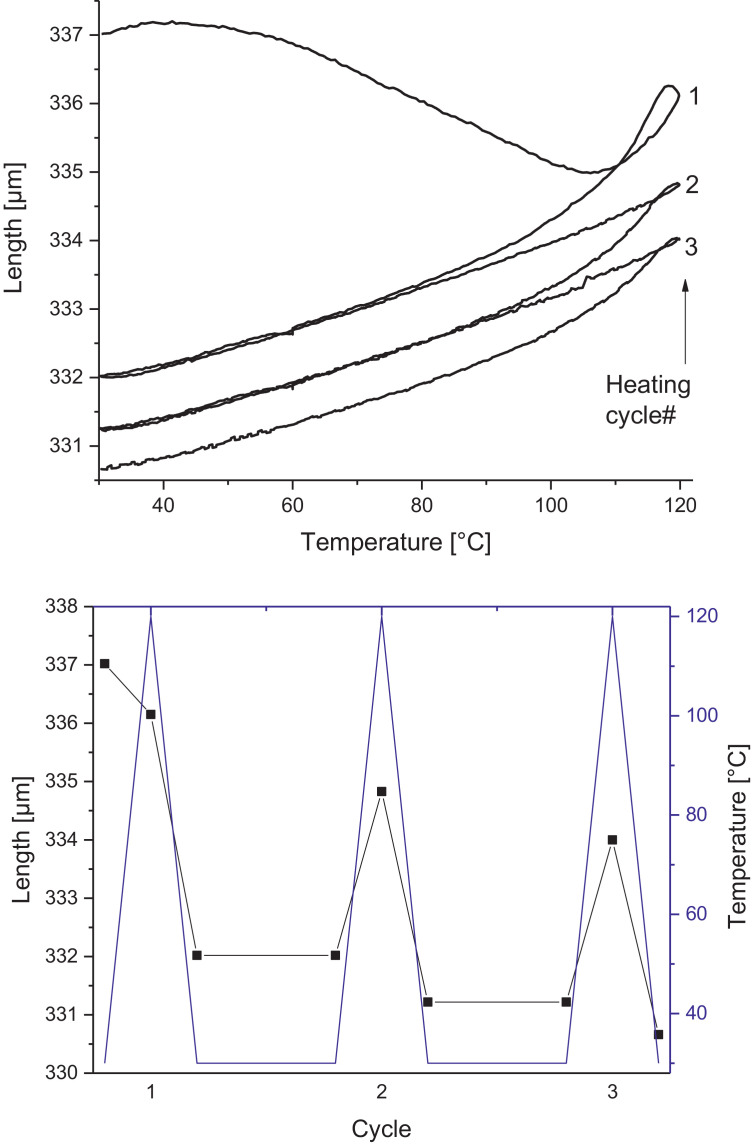
Left: Length vs. temperature of the upper envelope; right: sample length during the temperature programme, the temperature programme is plotted as blue line.

**Fig. 3:**
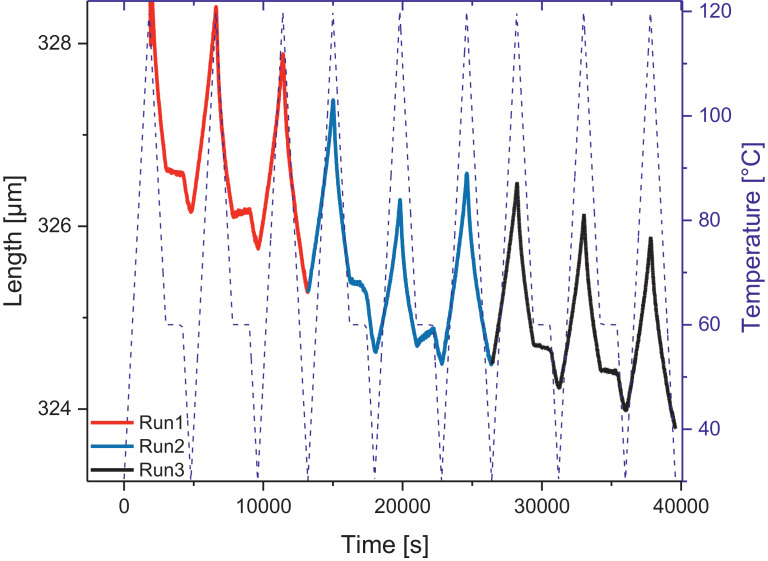
DL-TMA traces in three subsequent runs showing the upper envelope; temperature programme is plotted as blue dashed line.

**Fig. 4:**
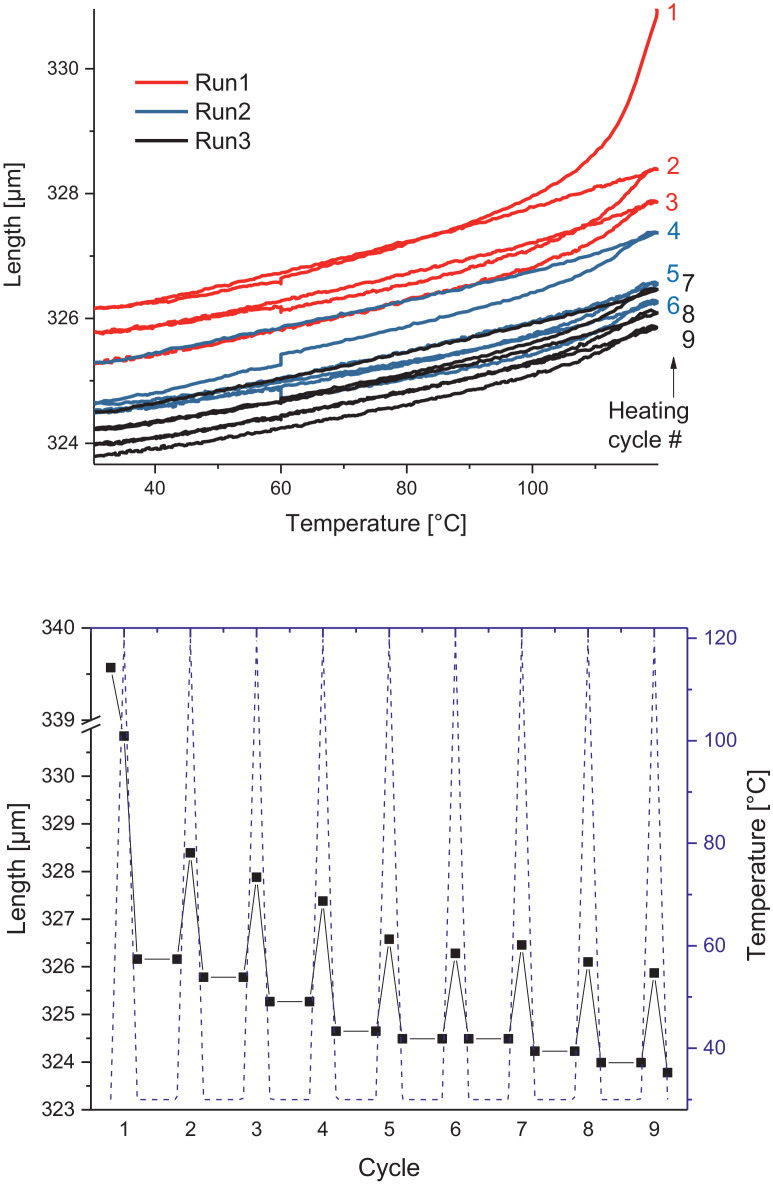
Left: Length vs. temperature of the upper envelope of the 3 runs; right: sample length during the temperature programme, the temperature programme is plotted as blue line.

**Fig. 5:**
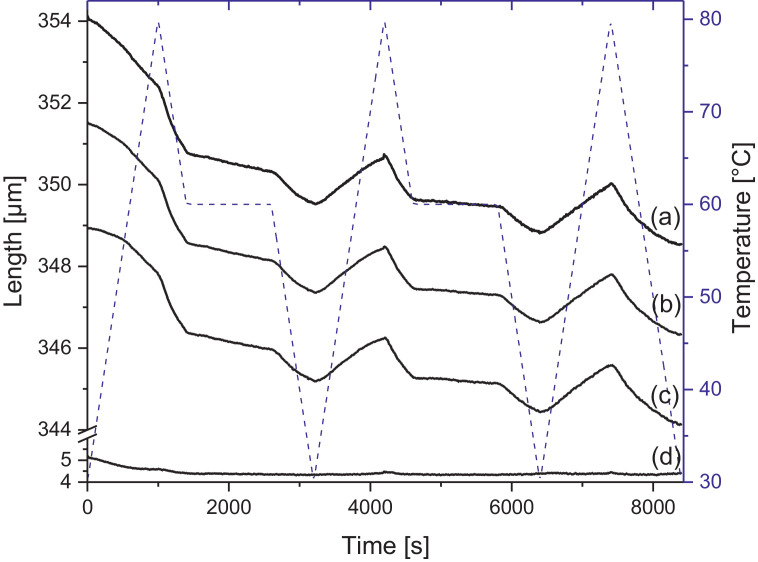
DL-TMA traces; (a) upper envelope; (b) middle envelope, or average change in elongation; (c) lower envelope; (d) amplitude level; temperature programme is plotted as blue dashed line.

**Fig. 6:**
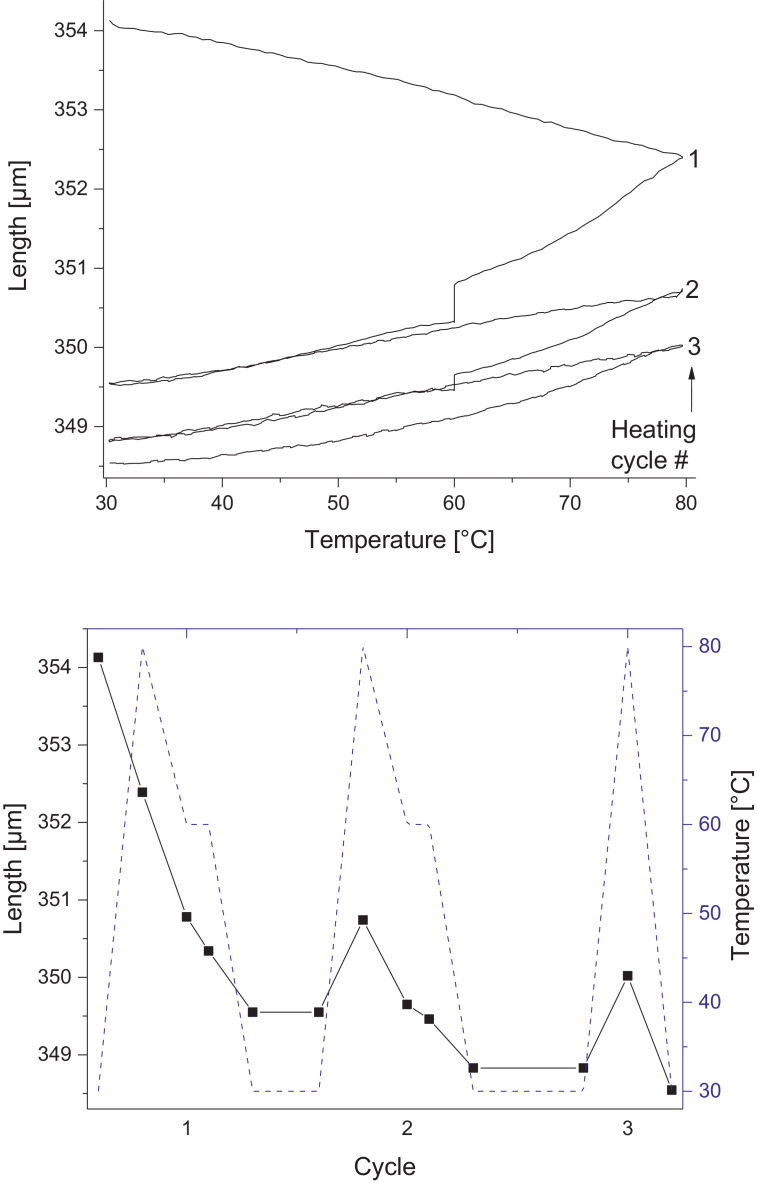
Left: Length vs. temperature of the upper envelope; right: sample length during the temperature programme, the temperature programme is plotted as blue line.

**Fig. 7:**
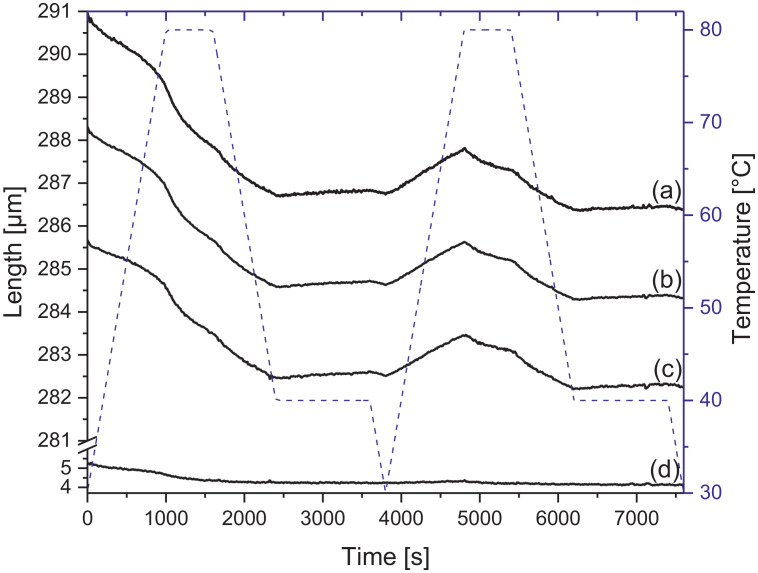
DL-TMA traces; (a) upper envelope; (b) average change in elongation; (c) lower envelope; (d) amplitude level; temperature programme is plotted as blue dashed line.

**Fig. 8:**
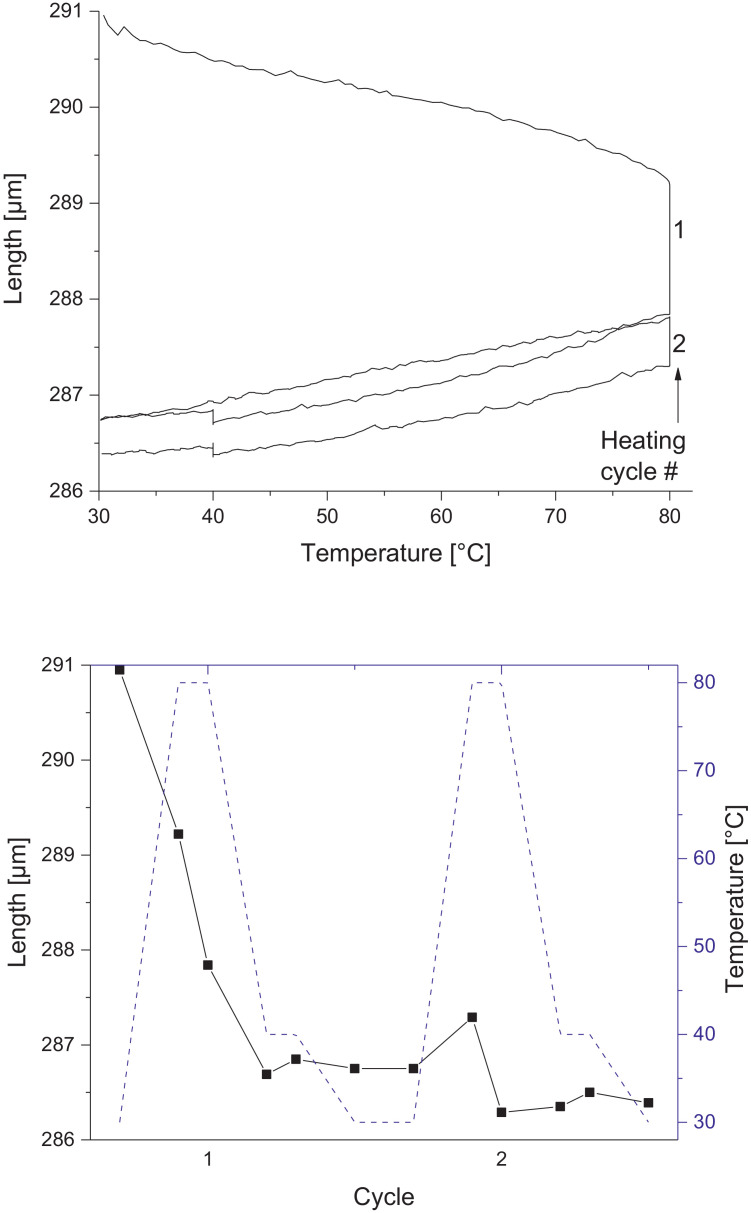
Left: Length vs. temperature of the upper envelope; right: sample length during the temperature programme, the temperature programme is plotted as blue line.

**Fig. 9:**
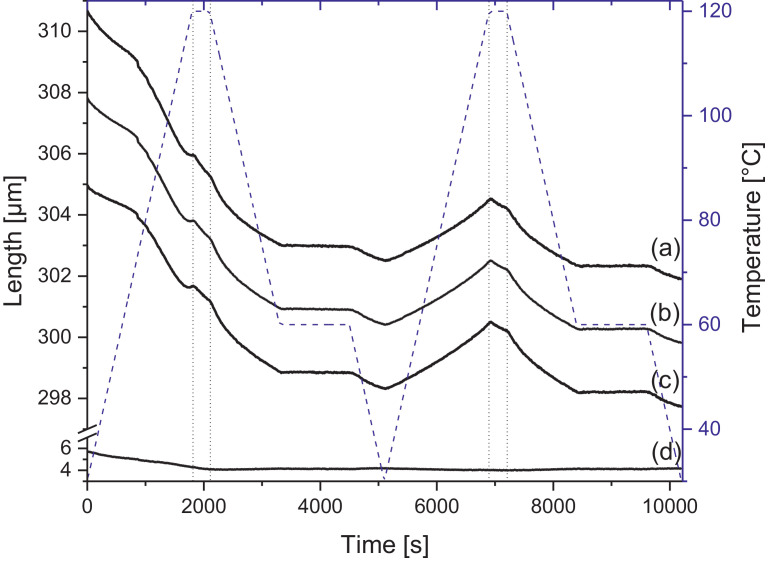
DL-TMA traces; (a) upper envelope; (b) average change in elongation; (c) lower envelope; (d) amplitude level; temperature programme is plotted as blue dashed line.

**Fig. 10:**
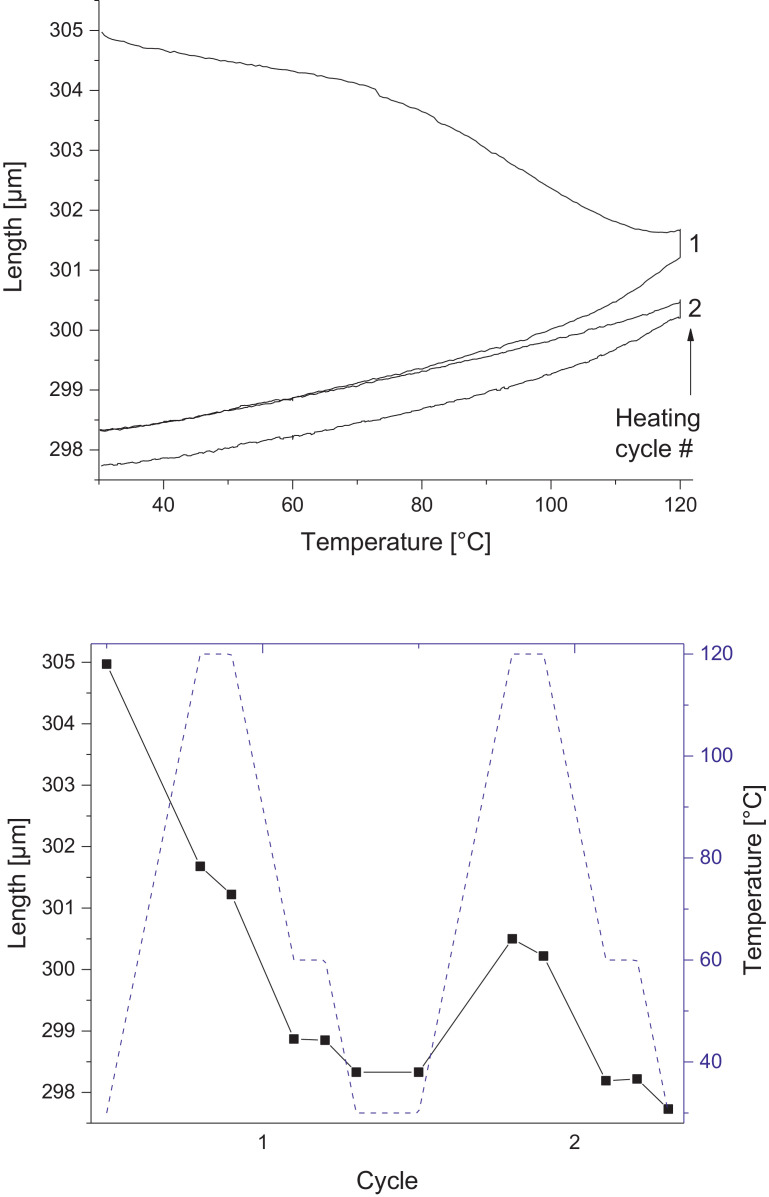
Left: Length vs. temperature of the upper envelope; right: sample length during the temperature programme, the temperature programme is plotted as blue line.

**Fig. 11:**
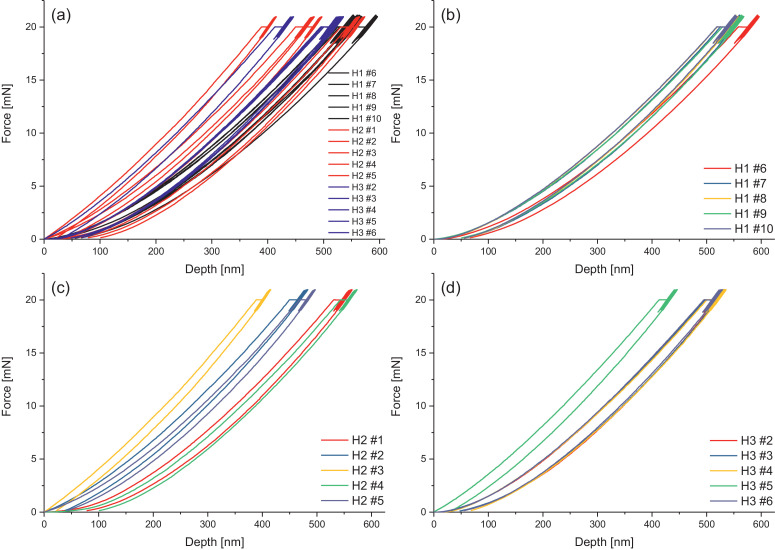
Nanoindentation measurements of 5 times repeated measurements of (a) sample DA resin all heating cycles; (b) of H1 sample DA resin after one healing cycle; (c) H2 sample DA resin after two healing cycles; (d) H3 sample DA resin after three healing cycles.

**Fig. 12:**
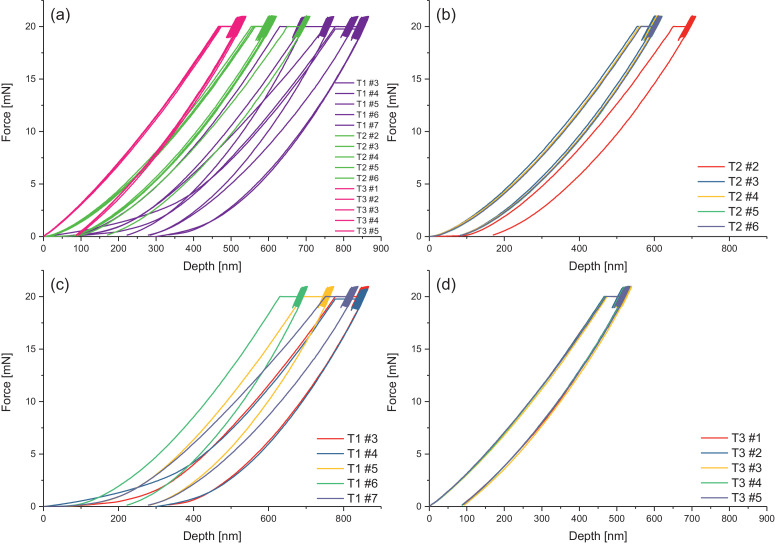
Nanoindentation measurements of 5 times repeated measurements of (a) sample DA resin all heating cycles at the rDA state; (b) of T1 sample DA resin at the first rDA reaction state; (c) T2 sample DA resin at the second rDA reaction state; (d) T3 sample DA resin at the third rDA reaction state.

Melamine-formaldehyde (MF) resins are duroplastic materials with good surface hardness, chemical stability and high transparency which makes them preferred surface finishing resins for paper-based decorative laminates [2]. Their excellent surface properties are due to their thermosetting character, which, on the other hand, is also responsible for the fact that self-healing of surface scratches is usually not possible with unmodified classic MF resins [3]. Recently, it has been shown that Diels Alder functional groups can be chemically integrated into melamine resin and that these groups are still reactive after condensation curing and can thermally cross-link reversibly [4], [5], [6]. It has also been shown that decorative laminates can be prepared with such DA modified resins that show self-healing activity when heated after being damaged by scratching [1]. The shown data are supplementary of the article Urdl et al. 2020 [1]. Fig. 1, Fig. 2, Fig. 3, Fig. 4, Fig. 5, Fig. 6, Fig. 7, Fig. 8, Fig. 9, Fig. 10 show the dynamic load thermomechanical profiles of cured MF resin film subjected to various temperature programs. Fig. 11 (a, b, c, and d) shows nanoindentation measurements of different chemical stages (rDA and DA) of DA resin during the self-healing cycle of the resin. Fig. 12 (a, b, c, and d) shows the nanoindentation measurements of the stages of the rDA reaction.

DL-TMA Measurements of DA Resin Applying Different Heating Programmes

Method 1: 30 to 120 °C (+3 °C min^−1^) – 120 to 60 °C (-3 °C min^−1^) – 60 °C isotherm 20 min–60 to 30 °C (-3 °C min^−1^) – 30 to 120 °C (+3 °C min^−1^) – 120 to 60 °C (-3 °C min^−1^) – 60 °C isotherm 20 min–60 to 30 °C (-3 °C min^−1^) – 30 to 120 °C (+3 °C min^−1^) – 120 to 30 °C (-3 °C min^−1^)

Method 2: 30 to 120 °C (+3 °C min^−1^) – 120 to 60 °C (-3 °C min^−1^) – 60 °C isotherm 20 min – 60 to 30 °C (-3 °C min^−1^) – 30 to 120 °C (+3 °C min^−1^) – 120 to 60 °C (-3 °C min^−1^) – 60 °C isotherm 20 min – 60 to 30 °C (-3 °C min^−1^) – 30 to 120 °C (+3 °C min^−1^) – 120 to 30 °C (-3 °C min^−1^) – repeated 2 times.

Method 3: 30 to 80 °C (+3 °C min^−1^) – 80 to 60 °C (-3 °C min^−1^) – 60 °C isotherm 20 min – 60 to 30 °C (-3 °C min^−1^) – 30 to 80 °C (+3 °C min^−1^) – 80 to 60 °C (-3 °C min^−1^) – 60 °C isotherm 20 min – 60 to 30 °C (-3 °C min^−1^) – 30 to 80 °C (+3 °C min^−1^) – 80 to 30 °C (-3 °C min^−1^)

Method 4: 30 to 80 °C (+3 °C min^−1^) – 80 °C isotherm (10 min) – 80 to 40 °C (-3 °C min^−1^) – 40 °C isotherm 20 min – 40 to 30 °C (-3 °C min^−1^) – 30 to 80 °C (+3 °C min^−1^) – 80 °C isotherm (10 min) – 80 to 40 °C (-3 °C min^−1^) – 40 °C isotherm 20 min – 40 to 30 °C (-3 °C min^−1^)

Method 5: 30 to 120 °C (+3 °C min^−1^) – 120 °C isotherm 5 min – 120 to 60 °C (-3 °C min^−1^) – 60 °C isotherm 20 min – 60 to 30 °C (-3 °C min^−1^) – 30 to 120 °C (+3 °C min^−1^) – 120 °C isotherm 5 min – 120 to 60 °C (-3 °C min^−1^) – 60 °C isotherm 20 min–60 to 30 °C (-3 °C min^−1^)

Nanoindentation Measurements of DA Resin Mimicing the Self-Healing Cycles

The nanoindentation traces of DA resin at different chemical stages (rDA and DA) during the self-healing cycles are presented in the following graphs. Fig. 11: shows the Nanoindentation measurements of the stages of DA reaction.

Fig. 12: shows the Nanoindentation measurements of the stages of rDA reaction, after heating the sample at 120 °C for 5 minutes and rapidly cooling to room temperature to freeze the rDA state.

## 2 Experimental Design, Materials and Methods

### 2.1 Materials

2-aminomethyl furan and 2-chloro-1,3,5-triazine-2,4-diamine were supplied from Merck KGaA (Darmstadt, Germany). Sodium bicarbonate, formaldehyde solution (37 wt.-%), N,N-dimethylformamide and ethanol (70%) were supplied from Carl Roth GmbH & Co. KG (Karlsruhe, Germany). Polyphenylmethane bis-maleimide (BMI) was supplied from HOS-Technik GmbH (Austria). All chemicals were used without further purification.

### 2.2 Methods

#### 2.2.1 Monomer preparation

Preparation of N^2^-(furan-2-ylmethyl)-1,3,5-Triazine-2,4,6-triamine (Fu-Mel) Monomer

The Fu-Mel monomer was synthesized based on a method that was earlier presented by us [5,6]. 50 mmol of 2-aminomethyl furan (Fu), 48 mmol of 2-6-chloro-1,3,5-triazine-2,4-diamine (Mel), and 48 mmol of NaHCO_3_ were combined with 40 mL of deionized H_2_O and 55 mL of EtOH. The reaction mixture was heated to 85 °C and kept at this temperature for 8.5 h. After the reaction was finished, EtOH was evaporated by applying vacuum. The reaction product had formed a yellowish precipitate. It was filtered from the aqueous solution and thoroughly washed with deionized water. The final reaction product was dried under vacuum overnight. No further purification procedure was applied to the Fu-Mel monomer. It was subsequently used for chemical transformation.

Preparation of monomeric Diels-Alder Adduct

The Diels-Alder reaction between Fu-Mel monomer and BMI to give the monomeric DA adduct was carried out using the basic procedure described in research papers published earlier on the synthesis of Fu-Mel-based particles [5,6]. 9 mmol BMI and 18 mmol Fu-Mel were dissolved in a total volume of 35 mL of dimethylformamide. The reaction solution was stirred and kept under N_2_ for 26 h at a constant reaction temperature of 60 °C.

#### 2.2.2 Polymer Preparation

Preparation of the DA-Pre-Polymer used for Diels-Alder Resin Synthesis

Preparation of the pre-polymer was performed as follows: 2 or 4 equivalents of formaldehyde were added to the DA adduct in dimethylformamide. The mixture was thoroughly mixed and kept at a reaction temperature of 70 °C. The reaction time was 40 min. The pre-polymer was dried at 65 °C for 12 h after synthesis.

Curing of DA Resin

Curing of the resin samples was brought about in a hot-press. The pre-polymer was hot pressed in a SUT-PUK 1600 laboratory press (Svoboda Umformtechnik GmbH, Vienna, Austria). A high gloss pressing plate was applied in the pressing step to provide for smooth surface appearance. Pressing was done at a constant pressure of 20 N cm^−2^. All samples subjected to a temperature profile consisting of four phases: (1) Heating phase with a temperature gradient from 65 to 120 °C within 200 s, (2) isothermal phase at 120 °C for 300 s, (3) cooling phase with a temperature gradient from 120 °C to 65 °C within 200 s, and (4) isothermal phase at 65 °C for 900 s.

#### 2.2.3 Characterization of DA resin

Dynamic Load Thermomechanical Analysis (DL-TMA)

DL-TMA was applied to characterize the curing state of the DA resin. A circular sample (diameter: 3 mm^2^) was punched and analysed applying a dilatometric mode with an oscillating force ranging from 0.1 to 0.5 N in a rectangular mode with a time period of 12 s. The temperature programme was selected such as to cover several heating cycles. Heating rate was maintained at 3 °C min^−1^. The detailed temperature programmes applied during the DL-TMA measurement procedure for the various samples were as follows:

*Sample DA01:* (a) temperature rise from 30 to 80 °C using a heating rate of +3 °C min^−1^, (b) temperature drop from 80 to 60 °C using a cooling rate of -3 °C min^−1^, (c) isothermal phase at 60 °C for a time period of 20 min, (d) temperature drop from 60 to 30 °C using a cooling rate of-3 °C min^−1^). This was repeated twice.
*Sample DA02:* (a) temperature rise from 30 to 80 °C using a heating rate of +3 °C min^−1^, (b) isothermal phase at 80 °C for 10 min, (c) temperature drop from 80 to 40 °C using a cooling rate of -3 °C min^−1^, (d) isothermal phase at 40 °C for 20 min, (e) temperature drop from 40 to 30 °C using a cooling rate of -3 °C min^−1^. This was repeated once.
*Sample DA03:* (a) temperature rise from 30 to 120 °C with a heating rate of +3 °C min^−1^, (b) temperature drop from 120 to 60 °C using a cooling rate of -3 °C min^−1^, (c) isothermal phase at 60 °C for 20 min, (d) temperature drop from 60 to 30 °C using a cooling rate of -3 °C min^−1^. This was repeated twice.
*Sample DA04:* (a) temperature rise from 30 to 120 °C with a heating rate of +3 °C min^−1^, (b) isothermal phase at 120 °C for 5 min, (c) temperature drop from 120 to 60 °C using a cooling rate of -3 °C min^−1^), (d) isothermal phase at 60 °C for 20 min, (e) temperature drop from 60 to 30 °C using a cooling rate of -3 °C min^−1^. This was repeated once.
*Sample DA05:* (a) temperature rise from 30 to 120 °C with a heating rate of +3 °C min^−1^, (b) temperature drop from 120 to 60 °C using a cooling rate of -3 °C min^−1^, (c) isothermal phase at 60 °C for 20 min, (d) temperature drop from 60 to 30 °C using a cooling rate of -3 °C min^−1^. This was repeated 8 times.

The Figures given in the manuscript are based on the raw data files DL-TMA_Method_1_FU_517_R92-Harz_a.csv (Sample DA01, Figs. 1 and 2), TMA_Method_2_FU_517_R92-Harz_run1.csv, TMA_Method_2_FU_517_R92-Harz_ run2.csv, TMA_Method_2_FU_517_R92-Harz_ run3.csv (Sample DA02, Figs. 3 and 4), TMA_Method_3.csv (Sample DA03, Figs. 5 and 6), TMA_Method_4.csv (Sample DA04, Figs. 7 and 8), TMA_Method_5.csv (Sample DA05, Figs. 9 and 10)

The upper, middle and lower envelope regard the range of elongation the material experiences during the dynamic load experiments. "Lower envelope" regards the lowest values measured for the dimension of the tested specimen in dependence of temperature during the dynamic load experiment, "high" envelope regards the largest values. "Middle envelope" represents the average dimension during the test in dependence of temperature and is, therefore, also called the “average change in elongation”.

Nanoindentation Measurements

A UNHT^3^ nanoindenter (Anton Paar, Graz, Austria) was used to characterize the cured DA resin. The device was equipped with a sphero-conical intender tip (100 µm, 90 °). All measurements were performed at room temperature. Measurements were done by applying a sinus on load mode with a constant strain rate to a maximum of 40 mN, a pause of 10 s, sinus frequency of 5 Hz and amplitude of 4 mN. Sinus on hold measurements were performed in a 2 × 2 matrix acquiring 4 measurements at a constant strain with a maximum load of 20 mN, a pause of 30 s and a sinus pause of 30 s with 1 Hz and 2 mN. The stiffness threshold was set to 150 µN µm^−1^. The approach distance to 3 µm and speed to 2 µm min^−1^.

The static Young's modulus was determined in phase 2 of the measurement under constant force using equation (1):

(1) $$E=\frac{\sigma }{\varepsilon }=\;\frac{F\;/\;A}{\Delta L\;/\;L_{0}}$$

In equation (1), σ means the mechanical stress acting on the specimen, expressed as the effective force F per area A, and ε means the strain or relative change in dimension ΔL induced in the sample in relation to its initial length ($L_{0}$).

By applying a sinusoidal indentation stress under a constantly high load force, the nanoindentation measurement is operated in a mode that corresponds to a dynamic mechanical analysis. Thus, the loss factor, tanδ, can be determined from the frequency dependent modulus $E_{IT}$. For this, the following relationships were used:

(2) $$E_{IT}=\;\sqrt{{E_{storage}}^{2}+\;{E_{loss}}^{2}}$$

(3) $$\tan\delta =\;\frac{E_{loss}}{E_{storage}}$$

where $E_{IT}$ denotes the frequency dependent modulus,$\;E_{storage}$ the storage modulus, i.e. the ability of the material to absorb energy, and$E_{loss}$ the loss modulus.

To measure different healing stages during the temperature treatment in the self-healing process, the DA resin was tempered at 120 °C for 5 min, then rapidly cooled down to room temperature to freeze the rDA state, and subsequently measured. Afterwards, the sample was again tempered at 60 °C in the oven in order to support DA reaction. Thereafter, the sample was measured again. The described procedure was repeated three times to measure the material properties during three heating (i.e., healing) cycles.

Figs. 11 and 12 given in the manuscript are based on the attached raw data files. Fig. 11 is based on the files Nanoindent_H1_curves, Nanoindent_H2_curves, and Nanoindent_H3_curves. Fig. 12 is based on Nanoindent_HT1_curves (Fig. 12b), Nanoindent_HT1b_curves (Fig. 12c), and Nanoindent_HT1a_curves (Fig. 12d). Fig. 12a shows all nanoindentation data shown in b, c, and d together in one graph for better direct comparison.

## Ethics Statement

This work did not involve human subjects or animal experiments.

## Declaration of Competing Interest

The authors declare that they have no known competing financial interests or personal relationships which have, or could be perceived to have, influenced the work reported in this article.

## Appendix Supplementary Materials

Image, application 1

Image, application 2

Image, application 3

Image, application 4

Image, application 5

Image, application 6

Image, application 7

Image, application 8

Image, application 9

Image, application 10

Image, application 11

Image, application 12

Image, application 13
